# Geraniin Ameliorates Hypertensive Vascular Remodelling in a Diet-Induced Obese Animal Model through Antioxidant and Anti-Inflammatory Effects

**DOI:** 10.3390/nu15122696

**Published:** 2023-06-09

**Authors:** Boon Hee Goh, Hong Sheng Cheng, Pricilla Tracy A/P A. Alexandra, Kang-Nee Ting, Uma Devi Palanisamy, Joash Ban Lee Tan

**Affiliations:** 1 School of Science, Monash University Malaysia, Bandar Sunway 47500, Malaysia; boon.goh@monash.edu; 2 Lee Kong Chian School of Medicine, Nanyang Technological University, Singapore 637551, Singapore; hscheng@ntu.edu.sg; 3 Faculty of Medicine, Segi University, Kuala Lumpur 50010, Malaysia; 4 School of Pharmacy, University of Nottingham Malaysia Campus, Semenyih 43500, Malaysia; kang-nee.ting@nottingham.edu.my; 5 Jeffrey Cheah School of Medicine and Health Science, Monash University Malaysia, Subang Jaya 47500, Malaysia; umadevi.palanisamy@monash.edu

**Keywords:** ellagitannin, high blood pressure, vascular remodelling, oxidative stress, vasodilation

## Abstract

Geraniin, an ellagitannin, has shown a potent blood pressure-lowering effect in vivo. Therefore, this study aims to further characterize the ability of geraniin to attenuate hypertensive vascular dysfunction, a key feature of cardiovascular disease (CVD) development. Hypertension was induced in male Sprague-Dawley rats through feeding a high-fat diet (HFD) for eight weeks, followed by oral administration of 25 mg/kg/day geraniin for four weeks. The parameters of vascular dysfunction such as the structure and function of blood vessels as well as the vascular oxidative stress and inflammation were evaluated. The outcomes of geraniin-treated rats were compared with those of untreated rats on either a normal diet (ND) or HFD and with HFD-fed rats treated with captopril (40 mg/kg/day). We found that geraniin supplementation effectively ameliorated HFD-induced hypertension and abnormal remodelling of the thoracic aorta by suppressing excessive vascular superoxide (O_2_^−^) radical generation and overexpression of pro-inflammatory mediators in the circulating leukocytes. Furthermore, compared to the ND-fed rats, geraniin also independently promoted the significant enlargement of the thoracic aortic lumen for blood pressure reduction. Notably, the vascular benefits of geraniin were comparable to that of captopril. Collectively, these data suggest that geraniin can mitigate hypertensive vascular remodelling caused by overnutrition, which potentially abrogates the further development of CVDs.

## 1. Introduction

Overnutrition is a risk factor that contributes to obesity, a significant feature that is commonly observed in patients with cardiovascular disease (CVD). CVDs are the leading cause of death worldwide [1]. In 2019, 17.9 million people died from CVDs, contributing to 32% of death globally. In general, obesity also brings about metabolic comorbidities such as hypertension, type II diabetes mellitus, and dyslipidemia, all of which can lead to CVDs [2]. Among these perturbations, hypertension is consistently observed in more than 60% of obesity cases [3]. Therefore, it has been suggested that obesity might be the most common cause of essential hypertension in modern society. Excessive dietary fat intake can lead to hypertension due to the hyperactivation of the renin-angiotensin-aldosterone system (RAAS) and the sympathetic nervous system (SNS) [4]. Hypertension and metabolic derangements can collectively cause blood vessel damage, which further provokes the development of CVDs [5].

Hypertension is strongly associated with vascular dysfunction. Elevated blood pressure is not only due to excessive vasoconstriction induced by the overactivation of RAAS and SNS but also pronounced oxidative stress levels in the vascular system, which in turn leads to endothelial dysfunction and vascular remodelling [5,6]. For instance, hyperglycemia and dyslipidemia can trigger the overexpression of vascular NADPH oxidase for superoxide (O_2_^−^) radical generation, leading to the repression of endothelial nitric oxide synthase (eNOS) expression, increased production of a potent vasoconstrictor called endothelin-1 (ET-1), and activation of inflammatory pathways [7,8,9]. As a result, these changes impair the ability of the endothelium to produce vasorelaxant factors such as nitric oxide (NO) and prostacyclin to mediate blood vessel relaxation. Furthermore, excessive production of vascular O_2_^−^ radicals also enhances the activation of matrix-metalloproteinase (MMPs) to mediate the degradation of the extracellular matrix (ECM) proteins (e.g., collagen and fibronectin) in the vessel wall, promoting the migration and proliferation of vascular smooth muscle cells (VSMCs) [6]. Consequently, this leads to the thickening of the arterial wall and reduces the lumen ratio of blood vessels, which are the two most common features observed in hypertensive vascular remodelling. An increase in media-to-lumen ratio subsequently results in both enhanced vascular elasticity and vascular stiffness. Moreover, prolonged feeding of high-caloric foodstuff can also encourage the overactivation of circulating leukocytes, causing the release of pro-inflammatory factors that can worsen vascular dysfunction [10]. Collectively, both endothelial dysfunction and vascular remodelling can cause an increase in peripheral resistance and elevated blood pressure, serving as a critical therapeutic target to improve the cardiovascular risk factors caused by obesity-induced hypertension.

Polyphenols are secondary metabolites produced in plants and have been long recognised for their beneficial effects in the management of various chronic health conditions, such as cancer, diabetes, obesity, neurodegenerative diseases, and CVDs [11]. Of particular interest is ellagitannin geraniin (or geraniin in short), which is a hydrolysable tannin under the large family of polyphenolic compounds. Geraniin can be found in several medicinal herbs such as *Geranium*, *Euphorbia*, and *Phyllanthus* spp., as well as in the rinds of *Nephelium lappaceum* (commonly known as rambutan) [12]. To our best knowledge, geraniin is most abundantly present in rambutan peels among all the plant species [13]. Our previous study showed that oral geraniin supplementation effectively improved various metabolic risk factors in a high-fat diet (HFD)-induced obese Sprague-Dawley (SD) rats, including hypertension, hyperglycemia, hypertriglyceridemia, central adiposity, and hepatic steatosis [14,15]. Our findings on geraniin’s anti-hypertensive effect are in good agreement with the other independent studies that employed spontaneously hypertensive rats (SHRs) [16,17]. Moreover, geraniin also successfully ameliorated the elevation of advanced glycation end products (AGEs), a metabolic factor that can potentially cause vascular dysfunction, and improved the circulating levels of various biomarkers of oxidative stress and inflammation in vivo, such as malondialdehyde (MDA), protein carbonyl content, myeloperoxidase activity, and interleukin-1β (IL-1β) [14,15,18]. However, no study has ever been conducted to evaluate the impact of geraniin on hypertensive vascular dysfunction. Therefore, this study aims to characterize the vascular benefits of geraniin, if any, in ameliorating endothelial dysfunction and vascular remodelling in male SD rats with HFD-induced obesity, a metabolic syndrome disease model that our group had previously established. Captopril, an angiotensin-converting enzyme (ACE) inhibitor that has shown excellent effectiveness in improving energy homeostasis and vascular dysfunction, was used as a positive control in our study to evaluate the performance of geraniin [19,20,21,22].

## 2. Materials and Methods

### 2.1. Geraniin Extraction and Purification

The extraction and purification of geraniin from the rinds of *Nephelium lappaceum* (rambutan) were performed according to a published method [13]. The high-performance liquid chromatography (HPLC) analysis indicated that the purity of the purified geraniin was >95% (Supplementary S1 Figure S1). The proton nuclear magnetic resonance (^1^H-NMR) and the negative ionisation mode of liquid chromatography-mass spectrometry (LC-MS) of the purified geraniin were consistent with the previous reports [13,23] (Supplementary S1 Table S1 and Figure S2). Geraniin crystals were collected and stored at −80 °C until use.

### 2.2. Animal Ethics and Housing Conditions

The use and handling of animals in this research were approved by the Monash University Animal Research Platform Animal Ethics Committees (AEC approval number: MUM/2018/09). Twenty-four post-weaning (3 weeks old) male Sprague-Dawley rats weighing 35 to 70 g were obtained from the Monash University Malaysia Animal Facility. The rats were housed individually in polypropylene cages containing highly absorbent bedding and were maintained on a 12-h light-dark cycle with a controlled temperature of (23 ± 1) °C in the animal housing facility. Food and distilled water were given ad libtium.

### 2.3. Diet Preparation, Composition, and Treatment

Hypertension was induced in the post-weaning rats through the feeding of purified ingredient-based high-fat diet (HFD) pellets. The preparation of the diet pellets was performed according to a published method [24] (Supplementary S2 Table S2). Briefly, a total of 24 rats were randomised into two groups, one receiving a normal diet (ND) (*n* = 6) and the other receiving an HFD (*n* = 18). The rats were maintained on diet feeding for 8 weeks. The food and water were replenished every day. The body weight was measured weekly while the food intake was recorded daily. After 8 weeks, the HFD group was further divided into 3 subgroups (*n* = 6) comprising vehicle control (10% glucose water), geraniin (25 mg/kg/day), and captopril (40 mg/kg/day; positive control). The dosage of geraniin was established based on an in-house pilot study showing that 25 mg/kg of geraniin was more effective to improve various metabolic derangements, including high blood pressure, compared to the higher dosages (50 and 100 mg/kg) [14]. The dosage of captopril was selected based on a previous study demonstrating that 40 mg/kg of captopril significantly improved energy balance and hypertension in rats with diet-induced obesity [19]. All the compounds were dissolved in 10% (*w*/*v*) glucose water prior to feeding to minimize animal resistance to the oral gavage [25]. The drugs were orally administered once daily in 10% glucose water using a gavage needle for a period of 28 days. At the endpoint of the experiment, the rats were fasted for 12 h prior to euthanasia by exsanguination. The rats were anaesthetised by the intraperitoneal injection of ketamine (75 mg/kg) and xylazine hydrochloride (10 mg/kg) before sacrificing with cardiac puncture to collect plasma and tissue specimens.

### 2.4. Blood Pressure Measurement

Systolic and diastolic blood pressure was measured with Mouse and Rat Tail Cuff Blood Pressure System (IITC Life Sciences, Los Angeles, CA, USA). The rats were placed into a plastic restrainer to restrict their movement throughout the measurement. A tail-cuff with a pulse transducer was applied to the tail of the restrained rats. The rat was then placed into a well-ventilated chamber equilibrated at 32 °C for 20–30 min to facilitate dilation of caudal arteries. Next, the triplicate readings of the systolic (SBP) and diastolic blood pressure (DBP) were recorded. The procedure was performed before the experiment (Week 0) and every week (Week 1–12).

### 2.5. Blood Plasma and Tissue Collection

A blood sample was collected in a vacutainer containing 0.5 M ethylenediaminetetraacetic acid (EDTA). Plasma was obtained by centrifugation of the blood samples at 4 °C, 2000× *g* for 20 min. The plasma supernatant was snap frozen in liquid nitrogen and stored at −80 °C until use. The thoracic aorta was excised, cleaned of surrounding fat and connective tissues, washed with ice-cold 1× phosphate-buffered saline (PBS), and cut into several 4 mm aortic rings. One of the aortic rings was fixed in 10% neutral buffered formalin for histology while the rest were either incubated in physiological Krebs solution at 37 °C for vasomotor assessment or snap-frozen in liquid nitrogen and stored at −80 °C until use. Furthermore, the retroperitoneal white adipose tissue (rWAT) was also excised and washed, followed by drying on an absorbent paper to remove excess fluids for weighing.

### 2.6. Isolation of Peripheral Blood Mononuclear Cells (PBMCs) from the Whole Blood Sample

PBMCs were isolated from the whole blood sample within 2–3 h after collection from rats. Briefly, six milliliters of whole blood sample were diluted with 1× PBS at a 1:1 ratio (*v*/*v*) and carefully layered onto Histopaque-1083 (Sigma Aldrich, St. Louis, MO, USA), followed by centrifugation at 400× *g* for 30 min with break off. The buffy coat was transferred to a new 15 mL falcon tube for washing with 1× PBS, followed by centrifugation at 250× *g* for 10 min. The washing step was performed twice. Next, the cell pellet was resuspended in 1 mL RPMI-1640 medium containing 10% foetal bovine serum (Sigma Aldrich, St. Louis, MO, USA). Ten microliters of cell suspension were used for cell counting with trypan blue stain (Sigma Aldrich, St. Louis, MO, USA) to assess the PBMC viability. The cell density of PBMCs was adjusted to 1 × 10^6^ viable cells/mL, followed by centrifugation to pellet the cells. Next, the cell pellet was resuspended in 1 mL of RNAlater (Sigma Aldrich, St. Louis, MO, USA) and stored at −80 °C until use.

### 2.7. Plasma Biochemical Assays

The transient and volatile nature of nitric oxide (NO) makes it unsuitable for most conventional detection methods [26]. However, two stable metabolites of NO, namely nitrate and nitrite, can be easily measured by colourimetric assays. Therefore, nitrate and nitrite are used as surrogate biomarkers of NO production in circulation. Briefly, the total nitrate/nitrite and endothelin-1 (ET-1) levels in the blood plasma were measured with the Nitric Oxide (Total) Detection kit (Enzo Life Sciences, Farmingdale, NY, USA) and Rat Endothelin-1 ELISA kit (FineTest, Wuhan, China), respectively, according to the manufacturer’s instructions. The assays were performed in duplicate. The colourimetric end points were measured with Infinite^®^ 200 PRO (TECAN, Zürich, Switzerland).

### 2.8. Vasomotor Assessment of the Thoracic Aorta

An aortic ring (4 mm) was hung between an L-shaped stick and a metal wire triangle connected to a force transducer (MLTF050/ST, AD Instruments, Oxford, UK) and incubated horizontally in a tissue chamber containing 10 mL physiological Krebs solution (119.78 mM NaCl, 5.37 mM KCl, 2.43 mM MgSO_4_, 1.18 mM KH_2_PO_4_, 25.24 mM NaHCO_3_, 1.26 mM CaCl_2_, and 11.77 mM glucose; pH 7.4) with a continuing supply of carbogen (95% O_2_ and 5% CO_2_) at 37 °C. Changes in muscle tension response produced by the tissue contraction and relaxation were recorded by a PowerLab data acquisition system (LabChart v7.3.4) and a computer (Hewlett-Packard, Palo Alto, CA, USA). The aortic ring was allowed to equilibrate for at least 30 min before the application of tension of 2 g wt. Another 30 min of equilibration was given after all the aortic rings were loaded with tension. The reactivity of tissues was examined by the addition of 60 mM KCl twice. Aortic rings that produced more than 40% of contraction in response to the high concentration of KCl were considered active. After that, the aortic rings were rinsed with Krebs solution and the tension was returned to baseline for 30–60 min before proceeding with the studies. For the vasoconstriction study, a cumulative concentration-response curve of phenylephrine (0.1 nM to 0.3 mM) was performed to investigate any change in the smooth muscle contractile responses caused by the activation of the α_1_-adrenergic receptor (Supplementary S3 Figure S3a) [27]. For the vasorelaxation study, the aortic ring was pre-contracted to at least 70% of maximal contraction with 0.1 µM phenylephrine before the cumulative addition of carbachol (0.1 nM to 0.3 mM)—a non-selective muscarinic receptor agonist that encourages the production of vasorelaxant factors such as NO and prostacyclin in the endothelium to mediate the relaxation of vascular smooth muscle layer (Supplementary S3 Figure S3b) [27]. The maximal relaxation (E_max_) and potency constant (pEC_50_) values were calculated using the GraphPad Prism Version 9 (San Diego, CA, USA) in which EC_50_ indicates the effective concentration of phenylephrine/carbachol that produces 50% of maximal aortic tissue response and pEC_50_ is taken as the negative common logarithm of EC_50_.

### 2.9. Histological Examination of the Thoracic Aorta

The well-fixed thoracic aortic ring specimens were subjected to conventional tissue processing and embedded in paraffin wax. Thin sections (5 µm) were produced and stained with hematoxylin and eosin (H&E) to visualize the tissue morphology. Nikon Eclipse Ti2 microscope was used to capture the microscopic images of the thoracic aortic rings (4× and 40×). The thickness of the aorta was measured with Nikon NIS-Element AR software (Version 5.1). The media thickness was determined by measuring the distance from the internal elastic lamina to the external lamina. For each slide, measurements from 4 points (12, 3, 6, and 9 o’clock positions) were averaged. The lumen inner diameter was determined from the average of 12 and 6, and 9 and 3 o’clock positions. The media-to-lumen ratio, an index of aortic vascular remodelling, was calculated based on the measured lumen inner diameter and media data [28].

### 2.10. Protein Extraction and Gelatin Zymography

Gelatin zymography was performed according to a published method with slight modifications to examine the matrix-metalloproteinase (MMP) activity in the thoracic aorta [28]. Briefly, the frozen thoracic aorta was homogenised in 3 volumes of ice-cold radioimmunoprecipitation (RIPA) buffer (150 mM NaCl, 1% Triton X-100, 0.5% sodium deoxycholate, 0.1% SDS, and 50 mM Tris, pH 8.0) containing fresh PMSF (Sigma Aldrich, St. Louis, MO, USA) and protease inhibitor cocktail (Roche, Basel, Switzerland), followed by incubation on ice for 10 min and centrifugation at 12,000× *g* for 15 min at 4 °C. The concentration of proteins present in the tissue homogenate was determined with the BCA assay kit (Nacalai Tesque, Kyoto, Japan) according to the manufacturer’s instructions. Next, ten micrograms of tissue homogenate were mixed with 5× non-reducing sample buffer and electrophoresed on a 7.5% SDS polyacrylamide gel containing 0.1% gelatin. After electrophoresis, the gel was rinsed with washing buffer for 1.5 h with gentle agitation at room temperature. The buffer was then changed to incubation buffer and incubated at 37 °C for 24 h. Gelatin gel was stained with Coomassie blue stain and then destained with 10% acetic acid, followed by image capture with G: Box Chemi XRQ system (Syngene, Cambridge, UK). The unstained area corresponds to the gelatin-digestive activity of MMPs. The gelatin digestion area was analysed with ImageJ software (Version 1.53t) and was normalised to that of β-actin expression as determined by Western blot.

#### Western Blot of β-Actin Expression

Ten micrograms of tissue homogenate were electrophoresed on a 7.5% SDS polyacrylamide gel and electro-transferred onto a nitrocellulose membrane (ThermoFisher Scientific, Waltham, MA, USA), followed by blocking with 5% skimmed milk in 1× Tris-buffered saline (TBS) containing 0.1% Tween-20 at room temperature for 2 h and incubated overnight at 4 °C with primary monoclonal antibody for β-actin (1:5000; Santa Cruz Biotechnology, Dallas, TX, USA). The membrane was washed with TBST 3 times for 10 min, followed by incubation with horseradish peroxidase goat anti-mouse IgG (1:3000; Santa Cruz Biotechnology, Dallas, TX, USA) at room temperature for 2 h. After washing the membranes, the blots were developed with Western Blotting Luminol Reagent (Santa Cruz Biotechnology, Dallas, TX, USA), and the antibody binding was detected by G: Box Chemi XRQ system (Syngene, Cambridge, UK). The band intensity of the β-actin protein expression was analyzed with ImageJ software (Version 1.53t).

### 2.11. Lucigenin-Chemiluminescence Assay

Lucigenin-chemiluminescence assay was performed according to a published method with slight modifications to examine the production of superoxide anion in the thoracic aorta [29]. Briefly, NADPH oxidase was extracted by homogenizing the frozen thoracic aorta in ice-cold lysis buffer (20 mM HEPES and 10 mM EDTA) containing fresh phenylmethylsulfonyl fluoride (PMSF) (Sigma Aldrich, St. Louis, MO, USA) and protease inhibitor cocktail (Roche, Basel, Switzerland) at a 1:10 ratio (*w*/*v*). The tissue homogenate was incubated on ice for 10 min, followed by centrifugation at 1000× *g* for 10 min at 4 °C. The protein concentration was determined with the bicinchoninic acid (BCA) assay kit (Nacalai Tesque, Kyoto, Japan) according to the manufacturer’s instructions. Next, fifty microliters of tissue homogenate were added to the Krebs-HEPES buffer (99 mM NaCl, 4.7 mM KCl, 1.2 mM MgSO_4_, 1 mM KH_2_PO_4_, 1.9 mM CaCl_2_, 25 mM NaHCO_3_, 11.1 mM glucose, and 20 mM HEPES, pH 7.4) containing 5 µM lucigenin, a concentration that does not appear to be involved in redox cycling [29]. The background luminescence signal was read every minute over 15 min at 37 °C with Infinite^®^ M200 (TECAN, Zürich, Switzerland). Next, the reaction was started by adding 1 mM NADPH substrate (Santa Cruz Biotechnology, Dallas, TX, USA) to the suspension (250 µL of final volume), followed by measurement of the luminescence signal for every 30 s over 20 min. Buffer blank was subtracted from each reading and the superoxide production was expressed as a relative light unit (RLU)/mg protein.

### 2.12. RNA Extraction and Real-Time PCR

Total RNA of the thoracic aorta and PBMCs was extracted using the RNeasy Fibrous Tissue Mini Kit (Qiagen, Mettmann, Germany). The concentration and purity of the RNA were determined by measuring the absorbance at 260 nm and 280 nm with a BioDrop spectrophotometer (Biochrom, Cambridge, UK). RNA integrity was examined with agarose gel electrophoresis to visualize the 18S and 28S ribosomal RNA (Supplementary S4 Figure S4). One microgram of total RNA was treated with the RNase-free DNase I (ThermoFisher Scientific, Waltham, MA, USA) prior to the cDNA synthesis which was carried out with the RevertAid First Strand cDNA synthesis kit (ThermoFisher Scientific, Waltham, MA, USA). Next, CFX96 real-time PCR system (Bio-Rad Laboratories, Hercules, CA, USA) was used to perform the SYBR green-based real-time PCR. The selected SYBR green master mix was SensiFAST SYBR green No-Rox kit (Bioline, London, UK). The target genes were inducible nitric oxide synthase (*iNOS*) and NADPH oxidase p47^phox^ subunit (*p47*) for the thoracic aorta, and *iNOS*, chitinase-3-like protein 1 (*Chi3l1*), integrin alpha X (*Itgax*), and *CD163* for PBMCs. Hypoxanthine phophoribosyl-transferase 1 (*Hrpt1*), succinate dehydrogenase complex flavoprotein subunit A (*Sdha*), and β-actin (*Bac*) were used as the endogenous reference genes for normalisation [15]. All primers were synthesised by First BASE Laboratories, Selangor, Malaysia. The nucleotide sequence of the forward and reverse primers, as well as the accession numbers, are outlined in Supplementary S4 Table S3. Relative expression of the genes of interest between the experimental groups was calculated using the 2^−ΔΔCq^ method.

### 2.13. Statistical Analysis

Statistical analysis was performed with GraphPad Prism Version 9 (San Diego, CA, USA). Data are expressed as the mean ± standard deviation (SD). Analysis of dependent variables with repeated measures such as cumulative weight gain, blood pressure, and food and calorie intake was performed using two-way analysis of variance (ANOVA) with repeated measures. The pairwise comparisons were performed with Bonferroni correction. Other variables were analyzed with one-way ANOVA followed by Tukey’s test. The level of statistical significance was set at *p* < 0.05.

## 3. Results

### 3.1. Geraniin Ameliorated Increase in SBP and DBP Induced by HFD

**Figure 1:**
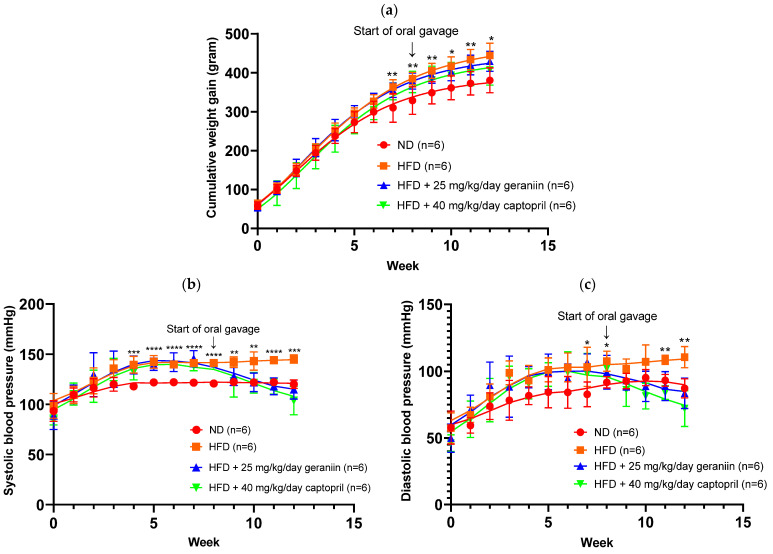
Weight gain and blood pressure parameters of the rats assigned to different treatment groups. (**a**) Cumulative weight gain, (**b**) SBP, and (**c**) DBP of the rats over 12 weeks. Data are expressed as mean ± SD with *n* = 6. * *p* < 0.05, ** *p* < 0.01, *** *p* < 0.001, and **** *p* < 0.0001 between the ND and HFD groups. SBP, systolic blood pressure; DBP, diastolic blood pressure; ND, normal diet; HFD, high-fat diet.

The obesogenic effect of HFD was observed to be statistically significant from Week 7 onwards (Figure 1a). Furthermore, compared to the ND-fed rats, HFD consumption also led to a marked increase in both SBP and DBP from Weeks 4 and 7 onwards, respectively (*p* < 0.05, Figure 1b,c).

The physiological parameters of the rats are summarised in Table 1. At the end of the experimentation period, HFD-fed rats exhibited a significantly higher body weight gain and rWAT to body weight ratio by 16.8% and 44.1%, respectively, compared to the ND group (*p* < 0.05, Table 1). The increased adiposity caused by HFD consumption was observed to be independent of calorie consumption (Table 1). Interestingly, captopril treatment led to a significant restriction of the daily calorie intake of the rats compared to both the ND and HFD groups (*p* < 0.05, Table 1). Although geraniin and captopril supplementation did not significantly reduce the total body weight and visceral fat accumulation of the high-fat diet-fed rats, both interventions effectively ameliorated the increase in SBP and DBP caused by an HFD consumption (*p* < 0.01, Table 1).

### 3.2. Geraniin Reversed HFD-Induced Vascular Remodelling

**Figure 2:**
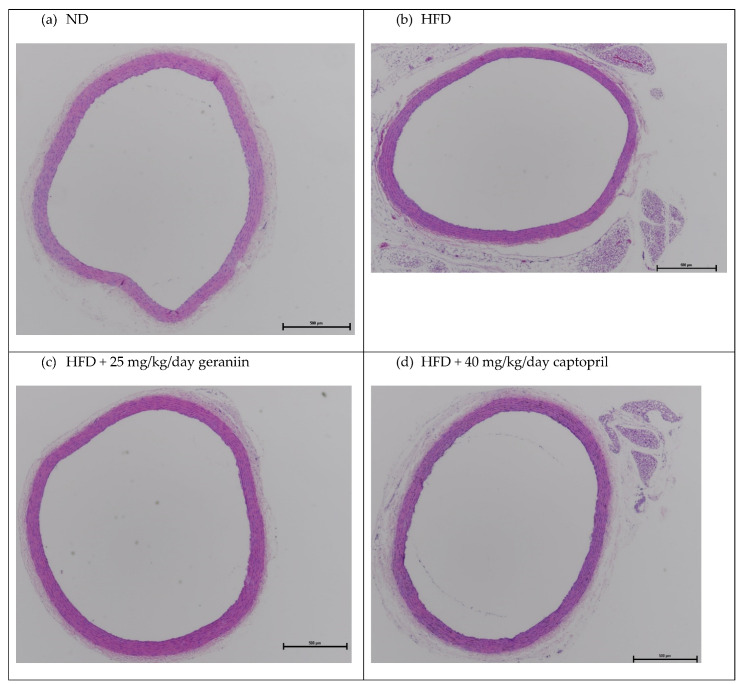
Representative images of the thoracic aorta sections stained with H&E staining at 4× magnification. ND, normal diet; HFD, high-fat diet.

**Figure 3:**
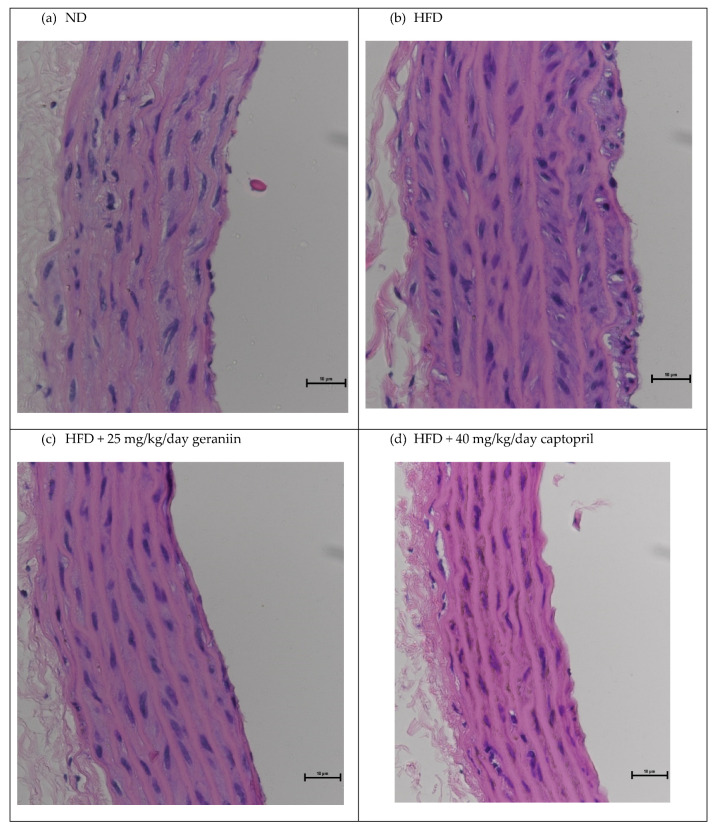
Representative images of the thoracic aorta sections stained with H&E staining at 40× magnification. ND, normal diet; HFD, high-fat diet.

**Figure 4:**
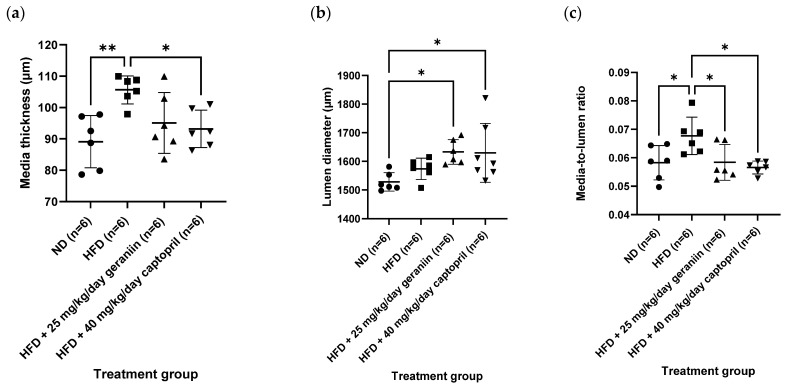
(**a**) The media thickness, (**b**) lumen diameter, and (**c**) media-to-lumen ratio of the thoracic aorta of rats assigned to different treatment groups. Data are expressed as mean ± SD with *n* = 6. * *p* < 0.05 and ** *p* < 0.01 compared between groups. ND, normal diet; HFD, high-fat diet.

The chronic effect of treatment regimens on the structure of the thoracic aorta was examined (Figure 2 and Figure 3). HFD feeding contributed to the significant thickening of the vessel wall by 18.5% compared to the ND group (*p* < 0.01, Figure 4a), which was successfully alleviated by captopril (*p* < 0.05, Figure 4a) but not geraniin. Interestingly, both geraniin and captopril significantly promoted the enlargement of the lumen diameter of the thoracic aorta by 6.80% and 6.61%, respectively, in comparison with the ND group (*p* < 0.05, Figure 4b). Moreover, as shown in Figure 4c, HFD consumption led to the remodelling of the thoracic aorta as evidenced by the significant increase in the media-to-lumen ratio by 16.2% compared to the ND group (*p* < 0.05). Conversely, the media-to-lumen ratio was effectively reduced following geraniin and captopril administration (*p* < 0.05, Figure 4c). Taken together, these findings indicate that geraniin could prevent the abnormal structural changes of the thoracic aorta in rats with hypertension induced by HFD. However, in this study, VSMC migration might not be involved in HFD-induced vascular remodelling because we found that all the treatment groups exhibited a similar extent of aortic MMP activities as each other (Supplementary S5 Figure S5).

**Figure 5:**
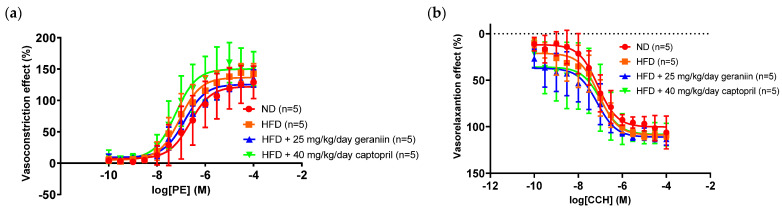
Concentration-response curve of (**a**) phenylephrine-induced vasoconstriction and (**b**) carbachol-induced vasorelaxation on the phenylephrine-induced pre-contracted aortic rings isolated from the rats assigned to different treatment groups. Data are expressed as mean ± SD with *n* = 5. PE, phenylephrine; CCH, carbachol; ND, normal diet; HFD, high-fat diet.

We next examined the impact of geraniin on the functional changes of blood vessels (if any) by evaluating the vascular reactivity of the thoracic aorta and the circulating levels of vasoactive substances. The aortic rings isolated from all the treatment groups demonstrated a concentration-dependent vasoconstriction and vasorelaxation response following the cumulative addition of phenylephrine and carbachol, respectively (Figure 5a,b). 

Pharmacological data such as the E_max_ and pEC_50_ of phenylephrine and carbachol were derived from Figure 5 and tabulated in Table 2. Of note, the contraction and relaxation responses of the thoracic aorta were not significantly altered by any treatment regimens (Table 2).

**Figure 6:**
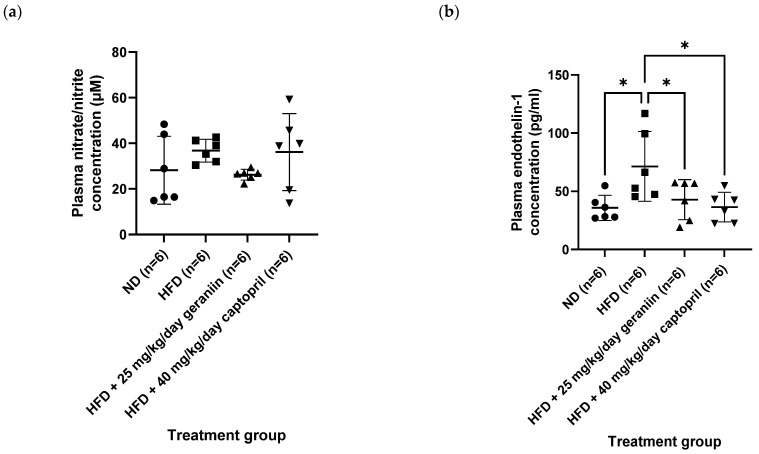
Blood plasma levels of (**a**) nitrate/nitrite and (**b**) ET-1 of the rats assigned to different treatment groups. * *p* < 0.05 compared to the HFD group. Data are expressed as mean ± SD with *n* = 6. ET-1, endothelin-1; ND, normal diet; HFD, high-fat diet.

In accordance with the insignificant change in the carbachol-induced vasorelaxation response, all the treatment groups also demonstrated comparable plasma nitrate/nitrite levels as each other (Figure 6a). Altogether, these findings suggest that HFD did not exacerbate the vasoconstriction response caused by α_1_-adrenergic receptor activation, nor impaired the endothelial function of the thoracic aorta for vasorelaxation. Nevertheless, we found that consumption of HFD elevated plasma ET-1 level by 99.4% relative to the ND group (*p* < 0.05, Figure 6b). This anomaly was effectively normalised by captopril (*p* < 0.05, Figure 6b) but not geraniin.

### 3.3. Geraniin Mitigated Excessive Superoxide Radical Production in the Thoracic Aorta and Systemic Inflammation Induced by HFD

**Figure 7:**
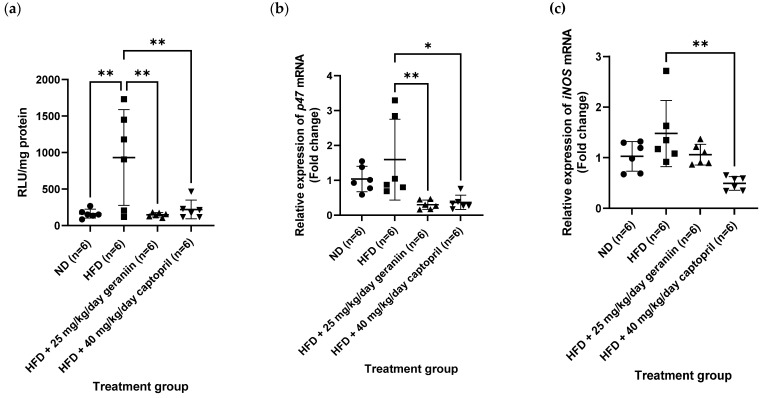
(**a**) The vascular O_2_^−^ radical level and the relative expression of (**b**) *p47* and (**c**) *iNOS* gene in the thoracic aorta of rats assigned to different treatment groups. *Hrpt1*, *Sdha*, and *Bac* were used as the endogenous reference genes. Data are expressed as mean ± SD with *n* = 6. * *p* < 0.05 and ** *p* < 0.01 compared to the HFD group. O_2_^−^, superoxide, RLU, relative luminescence unit; *p47*, NADPH oxidase p47^phox^ subunit; *iNOS*, inducible nitric oxide synthase; ND, normal diet; HFD, high-fat diet.

In our obese-hypertensive animal model, twelve weeks of HFD feeding significantly enhanced the generation of superoxide (O_2_^−^) radical in the thoracic aorta compared to the ND group (*p* < 0.01, Figure 7a), accompanied by a partial upregulation of the gene expression of NADPH oxidase p47^phox^ subunit (*p47*) and inducible nitric oxide synthase (*iNOS*) (Figure 7b,c, respectively). When the HFD-fed rats were treated with geraniin and captopril for a month, the aortic O_2_^−^ radical levels were markedly normalised and at comparable levels to the ND group (*p* < 0.05, Figure 7a). Furthermore, both geraniin and captopril also resulted in a significant downregulation of the gene expression of *p47* in the thoracic aorta compared to the HFD group (*p* < 0.05, Figure 7b). Furthermore, the captopril-treated rats also exhibited significantly lowered mRNA levels of *iNOS* in their aortic tissues compared to the HFD group (*p* < 0.01, Figure 7c).

**Figure 8:**
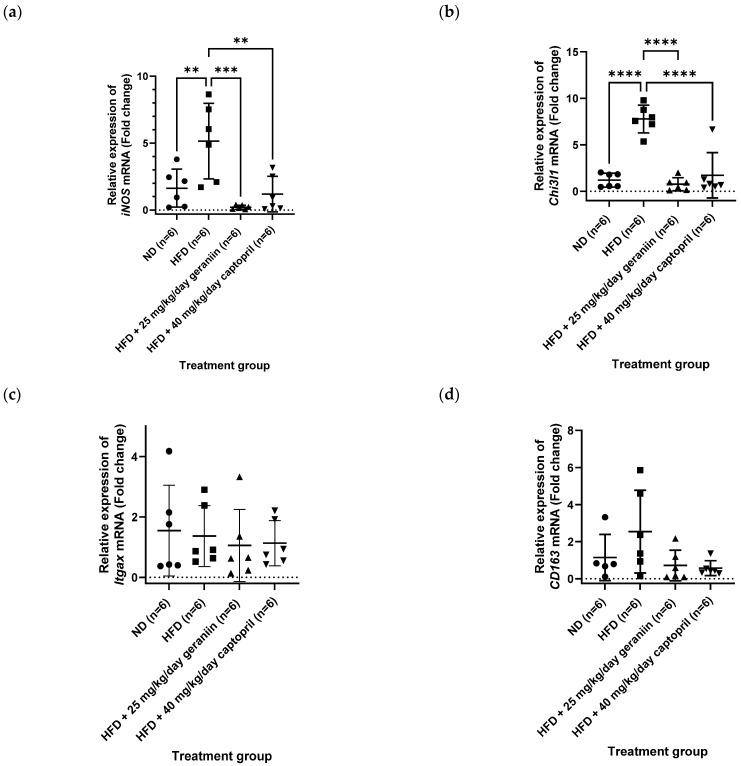
Relative expression of (**a**) *iNOS*, (**b**) *Chi3l1*, (**c**) *Itgax*, and (**d**) *CD163* in the PBMCs of rats assigned to different treatment groups. *Hrpt1* and *Bac* were used as the endogenous reference gene. Data are expressed as mean ± SD with *n* = 6. ** *p* < 0.01, *** *p* < 0.001, and **** *p* < 0.0001 compared to the high-fat diet group. PBMCs, peripheral blood mononuclear cells; *iNOS*, inducible nitric oxide synthase; *Itgax*, integrin subunit alpha X; *Chi3l1*, chitinase-3-like protein 1; ND, normal diet; HFD, high-fat diet.

Apart from the thoracic aorta, the effect of treatment regimens on the gene expression level of several pro-inflammatory mediators in the PBMCs was also investigated. Consumption of HFD led to the overexpression of *iNOS* and chitinase-3-like protein 1 (*Chi3l1*) gene compared to the ND group (*p* < 0.01, Figure 8a,b), which was effectively normalised by both geraniin and captopril (*p* < 0.01, Figure 8a,b). However, the gene expression of integrin alpha X (*Itgax*) was not significantly different in all the treatment groups (Figure 8c). Furthermore, although the HFD group demonstrated an increasing trend of *CD163* mRNA level compared to the ND group, the difference was not significant (Figure 8d). Likewise, geraniin and captopril also marginally downregulated the gene expression of *CD163* in the PBMCs compared to the HFD group (Figure 8d).

## 4. Discussion

This study demonstrated that chronic consumption of HFD in post-weaning SD rats led to central obesity, excessive intra-abdominal fat deposition, and hypertension. These metabolic derangements led to the onset of various vascular abnormalities, such as increased plasma level of ET-1, excessive production of O_2_^−^ radicals in the thoracic aorta, and overexpression of pro-inflammatory mediators in the PBMCs. These features are all associated with the abnormal remodelling of the thoracic aorta. However, HFD did not exacerbate the activities of MMPs, nor did it impair the endothelial function of the thoracic aorta as assessed by the endothelium-dependent vasorelaxation activity and circulating nitrate/nitrite levels when compared to the ND group. Interestingly, supplementation with geraniin did not significantly improve the adiposity parameters and plasma ET-1 level in HFD-fed rats. However, geraniin was able to alleviate hypertensive vascular remodelling, potentially by reducing aortic oxidative stress and systemic inflammation caused by HFD. Furthermore, geraniin was able to enlarge the lumen area of the thoracic aorta in HFD-fed rats, suggesting that it promoted blood vessel dilation in obese-hypertensive rats. This finding correlated with the observed reduction in SBP and DBP in geraniin-treated rats. Importantly, the vascular benefits of geraniin were found to be comparable to that of captopril, a commonly used blood pressure medication. Altogether, our present study revealed that geraniin supplementation was able to alleviate hypertensive vascular remodelling and promoted blood vessel dilation in obese-hypertensive rats, potentially preventing the onset of CVDs. These findings suggest that geraniin may be a promising natural compound for preventing vascular remodelling in obesity-related hypertension.

Endothelial dysfunction is one of the most common vascular dysfunction features found in many patients with metabolic syndrome [5]. In general, dysfunctional endothelium is characterised by the impairment of endothelium-dependent vasorelaxation, reduced NO bioavailability, and enhanced production of ET-1 [30,31,32]. Apart from mediating vasodilation, NO is also responsible for controlling the proliferation and migration of the vascular smooth muscle cells (VSMCs) in the blood vessels, which in turn prevents vascular remodelling [33]. ET-1 has the opposite action as NO and can be synthesised by both the endothelial cells and adipocytes upon stimulation by metabolic insults [34]. Although Munkong et al. (2016) showed that endothelial dysfunction could be successfully induced in vivo after one-month consumption of an HFD [35], we observed no significant change in the endothelial function in the rat’s thoracic aorta as well as the circulating nitrate/nitrite levels between the ND and HFD groups in our study. Notably, several animal studies supplemented the HFD-fed rodents with a high-fructose drink to induce endothelial dysfunction more effectively [36,37], thus suggesting that fructose may be the key ingredient that contributes to endothelial dysfunction. Therefore, further studies are required to optimize our purified ingredient-based HFD pellets to induce both endothelial dysfunction and metabolic syndrome in vivo for the mechanistic study of geraniin.

Although the consumption of HFD did not cause significant damage to the endothelial layer of the thoracic aorta, we observed that the HFD-fed rats exhibited vascular remodelling as evidenced by a significant increase in the aortic wall thickness and media-to-lumen ratio, the latter of which was effectively repressed by geraniin. Hypertension is strongly associated with the functional and structural alterations of the blood vessels as an adaptive response to increased intravascular pressure [6]. However, the remodelling may eventually become maladaptive and leads to vascular dysfunction. Vascular remodelling is a multifactorial process that includes leukocyte activation and enhanced liberation of reactive oxygen species (ROS) caused by metabolic factors such as ET-1, angiotensin II (Ang II), and advanced glycation end products (AGEs), which in turn promotes the change of the extracellular matrix (ECM) components in the blood vessel wall and encourages the proliferation and migration of VSMCs [28,38,39]. Our previous study showed that geraniin markedly normalised excessive circulating levels of AGEs in HFD-fed rats [15]. In the present study, the plasma ET-1 level was also marginally reduced by geraniin. Therefore, we speculate that the ability of geraniin to repress the onset of vascular remodelling caused by an HFD is, at least in part, due to the reduction of ET-1 and AGEs levels in the circulation.

Another interesting finding that we observed from the histological examination of the thoracic aorta is geraniin supplementation led to a significant enlargement of the aortic lumen in the HFD-fed rats compared to the ND group. This suggests that 4 weeks of oral intervention of geraniin might promote blood vessel dilation in obese-hypertensive rats, potentially reducing resistance to blood flow and ultimately leading to blood pressure reduction. Furthermore, the lack of significant change in the media-to-lumen ratio of the thoracic aorta between the ND and geraniin treatment groups suggests that the widening of blood vessel lumen induced by geraniin might be primarily due to its ability to relax the VSMCs within the vessel wall, rather than causing actual physical remodelling of the thoracic aorta. Notably, the vasodilatory effect of geraniin was probably independent of the NO and prostacyclin production because the carbachol-induced vasorelaxation response and the plasma nitrate/nitrite levels of the geraniin-treated rats were comparable to that of the ND and HFD groups. Therefore, we speculate that geraniin supplementation may promote blood vessel relaxation through other vasodilatory mechanisms such as the production of endothelium-derived hyperpolarizing factors (EDHFs) and/or inhibition of calcium mobilisation [40], which requires confirmation by further studies in the future.

As aforementioned, abnormal remodelling of blood vessels is often associated with pronounced oxidative stress in the vascular system. In this context, overexpression of NAPDH oxidase, especially its p47^phox^ subunit, is commonly reported in the vascular tissue of many hypertensive animal models [32,41,42]. NADPH oxidase is a multi-subunit enzyme complex that has been shown to be the primary source of ROS generation in the vascular system [43]. This ROS-producing enzyme can be activated by ET-1 and AGEs, which in turn leads to the induction of redox-sensitive nuclear factor-κβ (NF-κβ) signaling cascade to promote the expression of inducible NO synthase (*iNOS*), ET-1, and matrix-metalloproteinase (MMPs), all of which are implicated in the degradation and reorganisation of ECM components in the vessel wall as well as the proliferation and migration of VSMCs [44,45,46]. Furthermore, excessive intracellular ROS levels can also cause lipid peroxidation and protein oxidation, worsening blood vessel damage [43]. In the present study, the transcription of *p47* in the thoracic aorta of HFD-fed rats was significantly downregulated by geraniin, suppressing the liberation of the superoxide (O_2_^−^) radical exaggerated by an HFD in the thoracic aorta. Consistently, several studies also demonstrated that oral intervention of polyphenolic compounds such as curcumin, quercetin, and luteolin could improve endothelial dysfunction and vascular remodelling by inhibiting excessive ROS production in the blood vessels of hypertensive animal models [28,30,47].

Vascular remodelling is commonly associated with the overactivation of MMPs in the blood vessels [28]. MMPs are a class of zinc-dependent endopeptidases that mediate the degradation of the ECM components such as collagen and elastin in the vascular tissue to facilitate the rearrangement of VSMCs, which in turn provoke vascular remodelling. Since geraniin could improve the thickening of the aortic wall in our study, we next evaluated the effect of geraniin on the MMP activities in the thoracic aorta. In line with the previous studies, MMP-2 and MMP-3 activities were detected in the rat’s aortic tissue [28,48]. However, we found that all the treatment groups exhibited comparable MMP activities with each other. This suggests that the vascular remodelling observed in the HFD group was mainly attributed to the excessive proliferation of VSMCs without noticeable MMP activation for the rearrangement of the ECM scaffold in the vessel wall for VSMC migration.

Apart from vascular oxidative stress, systemic inflammation is also implicated in the pathogenesis of vascular remodelling [49]. PBMCs are a group of blood cells with round nuclei that encompass a heterogenous population of immune cells such as lymphocytes (70–90%), monocytes (10–30%), and dendritic cells (0.1–0.2%) [50]. It was reported that metabolic conditions could alter the immune cell population in animal models and human subjects. For instance, patients with type II diabetes mellitus exhibited the overexpression of various pro-inflammatory M1 polarisation markers [*CD16*, interleukin-6 (*IL-6*), *iNOS*, tumor necrosis factor-α (*TNF-α*), and *CD36*] in their PBMCs compared to healthy subjects [50]. In contrast, the mRNA transcript level of *CD163*, an anti-inflammatory M2 polarisation phenotype, was downregulated in the PBMCs of the diabetic subjects. In vivo, the SHRs exhibited a higher ratio of M1 to M2 macrophage population than the normotensive Wistar Kyoto (WKY) rats [51]. Furthermore, as compared to healthy individuals, patients with hypertension demonstrated a higher level of T helper 1 (Th1) and Th17 cells in their PBMCs, accompanied by increased circulating levels of interferon-γ (IFN-γ) and IL-17 [52]. Collectively, these findings indicate that the overactivation of the immune system may contribute to the progression of metabolic dysfunction, including hypertension, through the inflammatory pathways.

Our previous study showed that three months of consumption of an HFD resulted in a significant elevation of the circulating lipopolysaccharide (LPS)-binding protein level, which is an acute-phase protein that binds to the bacterial LPS, and a reduction of plasma IL-10 concentration; the former of which was effectively decreased following geraniin supplementation [15,53]. Of note, LPS is a bacterial toxin produced by gram-negative bacteria in the gut tissue, which is known to trigger the overactivation of leukocytes. Therefore, we hypothesised that geraniin might stabilize immune cell function exaggerated by an HFD, which in turn diminishes the onset of vascular inflammation. Indeed, we showed that geraniin markedly normalised the overexpression of *iNOS* and chitinase-3-like protein 1 (*Chi3l1*) caused by an HFD in the PBMCs comparable to that of the ND group. *iNOS* is a calcium-independent enzyme induced by cytokines or pathogenic stimuli such as LPS [54]. Under normal circumstances, immune cells express *iNOS* upon stimulation to generate NO to eliminate pathogens such as bacteria and viruses. However, prolonged activation of *iNOS* can enhance NO liberation in circulation, which in turn causes oxidative damage to various organ tissues, including the blood vessels. *Chi3l1* is a pro-inflammatory glycoprotein produced by Th17 cells and macrophages [55,56]. Several studies showed that *Chi3l1* can lead to vascular dysfunction by inducing a chemotactic effect on the endothelial and vascular smooth muscle layer of the blood vessels, as well as enhancing the migration and rearrangement of the endothelial cells and VSMCs in the vasculature [57,58]. Moreover, an augmented circulating level of *Chi3l1* is also associated with a higher prevalence of hypertension and atherosclerosis [59,60,61,62]. Taken together, we postulate that geraniin might protect against hypertensive vascular remodelling by attenuating the overexpression of *iNOS* and *Chi3l1* in the PBMCs.

Although our study provided evidence of the vascular benefits of geraniin in vivo, the oral bioavailability of this polyphenolic compound is rather poor because of its large molecular size, which does not facilitate intestinal absorption into circulation [63,64]. Upon oral ingestion, geraniin is broken down into various smaller metabolites by the gut microbes in the intestinal tissue for better absorption into the body. These metabolites include corilagin, ellagic acid, gallic acid, and various urolithin derivatives. Therefore, we believe that the bioactivities of geraniin observed in our present study are probably owed by its derived metabolites. Indeed, it has been shown that oral intervention of corilagin could prevent vascular remodelling in an atherosclerotic animal model by restraining the overactivation of aortic MMPs [65]. In vitro, ellagic acid and gallic acid treatment were found to suppress the excessive proliferation of VSMCs induced by oxidised LDL cholesterol and oleic acid, respectively [66,67]. Furthermore, ellagic acid also successfully normalised the overexpression of NADPH oxidase p47^phox^ subunit protein in the thoracic aorta of L-NAME-induced hypertensive rats [42]. In organ bath testing, ellagic acid and gallic acid were shown to attenuate excessive vasoconstriction caused by the incremental addition of CaCl_2_ through the inhibition of the L-type calcium channel [68,69]. Furthermore, gallic acid was also shown to promote the hyperpolarisation of the vascular smooth muscle layer by activating the voltage-dependent and inward rectifier potassium channels, which in turn promotes blood vessel relaxation [69]. Altogether, the antiproliferative, antioxidant, and vasodilatory effects of ellagic acid and gallic acid reported by previous studies coincide with our findings on the improvement of vascular remodelling and oxidative stress as well as the enlargement of the aortic lumen in the HFD-fed rats following geraniin supplementation. This suggests that the observed vascular benefits of geraniin in our study might be, at least in part, attributed to ellagic acid and gallic acid. Therefore, the integration of metabolomic analysis of geraniin-derived metabolites in circulation is warranted in the future to better decipher the pharmacological action of geraniin.

### Study Limitations

There are several shortcomings in our present study that need to be addressed in the future. Firstly, further optimisation of the induction of endothelial dysfunction is required for the mechanistic study of geraniin. Secondly, we did not evaluate the nuclear translocation of NF-κβ as well as the expression level of its other downstream targets such as cytokines and cell adhesion molecules [70] in the thoracic aorta due to financial constraints. Therefore, geraniin’s effects on vascular inflammation have yet to be fully explored. Last but not least, due to the heterogeneity of PBMCs, the impact of geraniin on the subtypes of the immune cell population remains to be elucidated. Therefore, further isolation of immune cells from the PBMC pool is warranted to provide us with a better understanding of the geraniin’s effect on the inflammatory stage of the different immune cell subtypes.

## 5. Conclusions

In conclusion, our findings demonstrate that oral administration of geraniin at a daily dosage of 25 mg/kg/day for one month successfully improved the abnormal remodelling of the thoracic aorta caused by an HFD, accompanied by the amelioration of vascular oxidative stress and systemic inflammation. Furthermore, geraniin also independently caused the enlargement of the lumen diameter of the thoracic aorta. Taken together, the beneficial effects of geraniin make it an interesting candidate for alleviating the development of hypertensive vascular remodelling similar to captopril. It is speculated that the observed bioactivities of geraniin may be attributed to its derived metabolites following oral ingestion. Therefore, a metabolomics study is warranted in the future to examine the circulating level of geraniin-derived metabolites to gain a better understanding of the pharmacological action of geraniin against diet-induced vascular dysfunction.

## Figures and Tables

**Table 1:** The physiological parameters of the rats assigned to different treatment groups.

Physiological Parameters	Treatment Group			
	ND	HFD	HFD + 25 mg/kg/day Geraniin	HFD + 40 mg/kg/day Captopril
Initial body weight (gram)	58.5 ± 9.3	62.2 ± 10.1	56.8 ± 11.5	57.8 ± 7.7
Final body weight (gram)	380.6 ± 31.9	444.4 ± 31.3 *	429.4 ± 25.7	407.4 ± 39.1
rWAT to body weight ratio (%)	2.54 ± 0.82	3.66 ± 0.42 *	3.02 ± 0.66	2.73 ± 0.41
Calorie intake (kcal/day)	60.8 ± 4.3	63.0 ± 6.0	60.0 ± 6.0	54.7 ± 4.1 *^, #^
SBP (mmHg)	120.2 ± 4.3	145.2 ± 4.1 ***	114.8 ± 9.5 ^####^	103.8 ± 14.1 *^, ####^
DBP (mmHg)	87.2 ± 7.1	110.5 ± 7.8 **	83.5 ± 11.4 ^##^	72.7 ± 14.1 ^####^

Data are expressed as mean ± SD with *n* = 6. * *p* < 0.05, ** *p* < 0.01, and *** *p* < 0.001 compared to the ND group. ^#^ *p* < 0.05, ^##^ *p* < 0.01, and ^####^ *p* < 0.0001 compared to the HFD group. ND, normal diet; HFD, high-fat diet; rWAT, retroperitoneal white adipose tissue; SBP, systolic blood pressure; DBP, diastolic blood pressure.

**Table 2:** Maximal tissue response and phenylephrine and carbachol drug potency on aortic rings isolated from the rats assigned to different treatment groups.

Pharmacological Parameters	Treatment Group			
	ND	HFD	HFD + 25 mg/kg/day Geraniin	HFD + 40 mg/kg/day Captopril
Vasoconstriction Emax (%)	125.1 ± 27.4	139.0 ± 13.2	127.8 ± 10.3	150.7 ± 31.5
Vasorelaxation Emax (%)	95.4 ± 4.5	108.9 ± 6.1	111.2 ± 4.1	108.3 ± 9.0
PE pEC50	6.55 ± 0.60	6.95 ± 0.42	6.70 ± 0.26	7.21 ± 0.35
CCH pEC50	7.34 ± 0.43	7.28 ± 0.77	7.27 ± 0.49	7.32 ± 0.86

Data are expressed as mean ± SD with *n* = 5. E_max_, the maximal response of tissue; PE, phenylephrine; CCH, carbachol; pEC_50_, potency constant; ND, normal diet; HFD, high-fat diet.

## Data Availability

Data are contained within the present article.

## Supplementary Materials

The following supporting information can be downloaded at: https://www.mdpi.com/article/10.3390/nu15122696/s1, Figure S1: HPLC chromatogram of the purified geraniin that showed a purity of 96.7%; Figure S2: Mass-to-charge ratio of the purified geraniin as determined by the negative ionisation mode of LC-MS; Figure S3: Representative isometric trace of (a) phenylephrine-induced aortic ring contraction and (b) carbachol-induced relaxation on the phenylephrine-induced pre-contracted aortic ring isolated from rats receiving a ND; Figure S4: Agarose gel image of the RNA samples extracted from the (a) thoracic aorta and (b) PBMCs of rats assigned to different treatment groups; Figure S5: The activities of MMPs in the thoracic aorta of rats assigned to different treatment groups; Table S1: 1H-NMR chemical shifts of the purified geraniin in comparison to the data published by [23]; Table S2: Macronutrient composition and ingredients of ND and HFD pellets [24]; Table S3: Accession numbers, forward and reverse primers of the endogenous reference and target genes as well as amplicon size of the PCR products.
