# Supplementary material for: Geraniin Ameliorates Hypertensive Vascular Remodelling in a Diet-Induced Obese Animal Model through Antioxidant and Anti-Inflammatory Effects
Source: Nutrients. 2023 Jun 9;15(12):2696. doi: 10.3390/nu15122696 (PMC10302501; doi:10.3390/nu15122696)
Supplement: Supplementary file 1 [file nutrients-15-02696-s001.zip › nutrients-2412898-supplementary.pdf]

Supplementary file

Supplementary S1: Geraniin extraction and purification

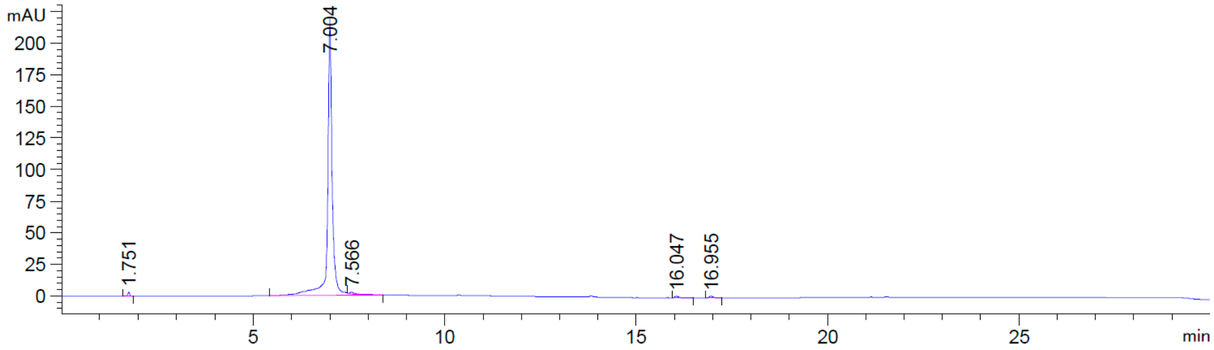

Figure S1. HPLC chromatogram of the purified geraniin that showed a purity of 96.7%.

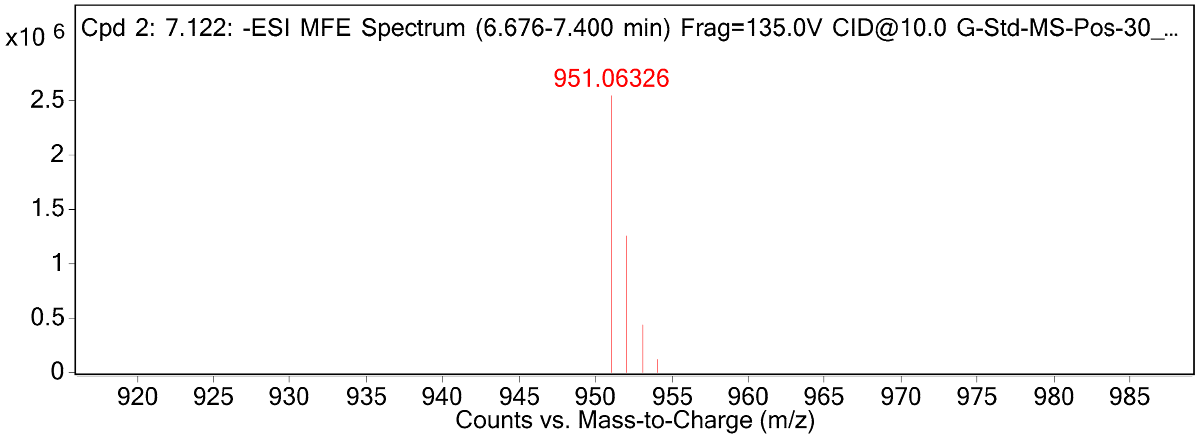

Figure S2. Mass-to-charge ratio of the purified geraniin as determined by the negative ionization mode of LC-MS.

Table S1. ^{1}H-NMR chemical shifts of the purified geraniin in comparison to the data published by [23].

Functional groups	^{1}H-NMR chemical shift (300 MHz, DMSO-d6)	
	Purified geraniin	[23]
Glucose		
1	6.36 (1H, d)	6.35 (1H, d)
2	5.33 (1H, d)	5.33 (1H, d)
3	5.40 (1H, s)	5.39 (1H, s)
4	5.21 (1H, s)	5.20 (1H, s)
5	4.70 (1H, t)	4.70 (1H, t)
6	4.38 (2H, m)	4.37 (2H, m)

Galloyl (Ring A)		
2 & 6	7.03 (2H, s)	7.03 (2H, s)
HDDP (Rings B & C)		
3 & 3′	6.46, 6.78 (1H, s)	6.45, 6.78 (1H, s)
DHHDP (Ring D)		
3	7.05 (1H, s)	7.05 (1H, s)
DHHDP (Ring E)		
1′	4.88 (1H, s)	4.88 (1H, s)
3′	6.36 (1H, s)	6.38 (1H, s)

Supplementary S2: Nutritional composition of the purified ingredient-based ND and HFD pellets

Table S2. Macronutrient composition and ingredients of ND and HFD pellets [24].

Macronutrient	Normal diet	High-fat diet
Protein (kcal%)	20	20
Carbohydrate (kcal%)	70	20
Lipid (kcal%)	10	60
Saturated (%)	36.6	57.9
Monounsaturated (%)	29.0	28.8
Polyunsaturated (%)	32.0	8.4
Trans (%)	1.8	3.6
Energy content (kcal/g)	3.9	5.3
Ingredient		
Casein	200	200
L-cystine	3	3
Corn starch	525.5	18
Maltodextrin	125	125
Sugar	50	50
Cellulose	50	50

Milk fat	20	245
Corn oil	25	25
AIN-93G Mineral mix	35	35
AIN-93-VX Vitamin mix	10	10
Choline bitartrate	2	2
t-butylhydroquinone	0.014	0.014

Supplementary S3: Vasomotor assessment of the thoracic aorta

(a)

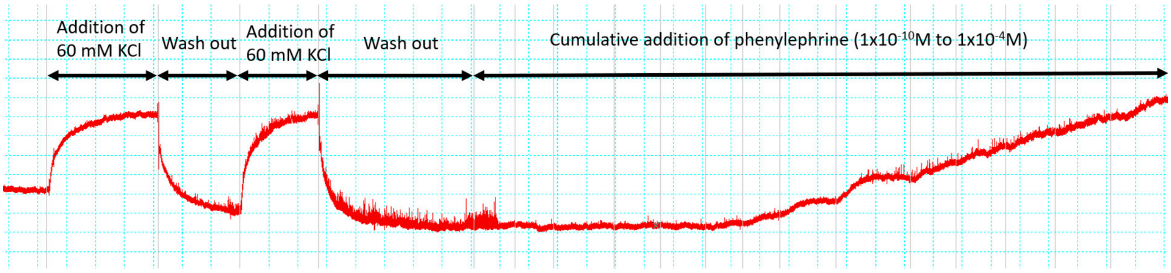

(b)

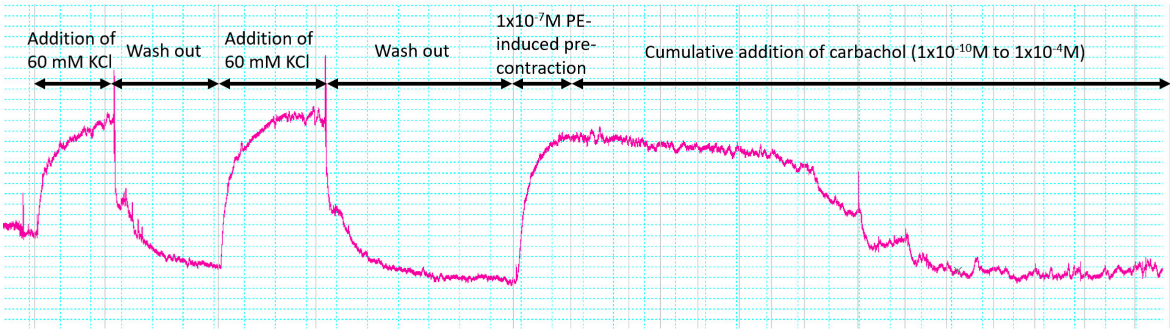

Figure S3. Representative isometric trace of (a) phenylephrine-induced aortic ring contraction and (b) carbachol-induced relaxation on the phenylephrine-pre-contracted aortic ring isolated from rats receiving a ND.

Supplementary S4: RNA extraction and real-time PCR

(a)

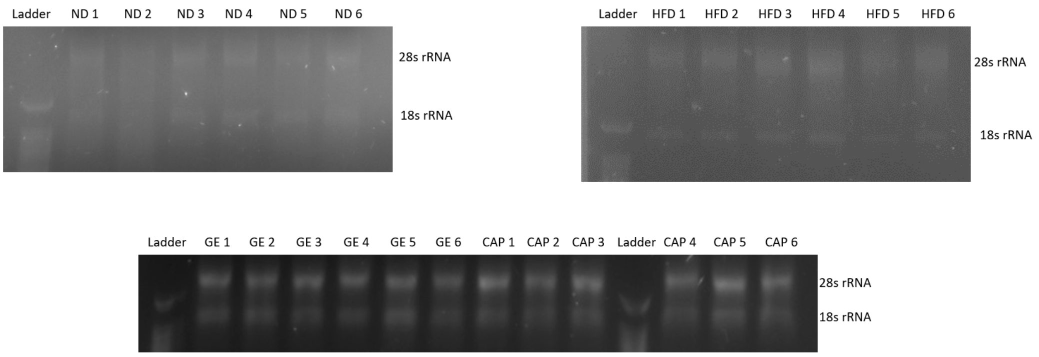

(b)

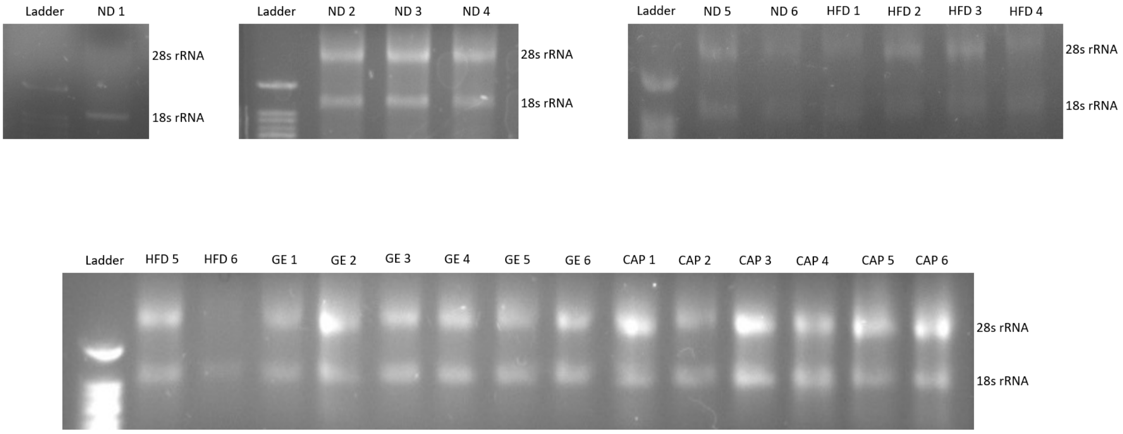

Figure S4. Agarose gel image of the RNA samples extracted from the (**a**) thoracic aorta and (**b**) PBMCs of rats assigned to different treatment groups. Two clear RNA bands are 18S (1.9 kb) and 28S (4.7 kb) ribosomal RNA (rRNA). The samples were run in a 1% agarose gel (0.5X TBE buffer) at 50V for 45 mins. ND, normal diet; HFD, high-fat diet; GE, geraniin; CAP, captopril.

Table S3. Accession numbers, forward and reverse primers of the endogenous reference and target genes as well as amplicon size of the PCR products.

Target gene	Accession number	Nucleotide sequence (5′ → 3′)		Amplicon size (bp)
		Forward primer	Reverse primer	
*Bac**	NM_031144	GTA TGG GTC AGA ACG ACT CC	GTT CAA TGG GGT ACT TCA GG	81
*Hrpt1**	NM_012583	CTG GAA AGA ACG TCT TGA TTG	GTA TCC AAC ACT TCG AGA GG	146
*Sdha**	NM_130428	GGC TTT CAC TTC TCT GTT GG	CCA CAG CAT CAA ACT CAT GG	103
iNOS	NM_012611	GAC TGG ACT TTT AGA GAC GCT T	TGT CTG TGA CTT TGT GCT TCT	116

p47	NM_053734	ACA CCT TCA TTC GCC ACA TC	GTA GAC CAC CTT CTC CGA CA	116
Chi3l1	NM_053560	CTG AGC AGG AGT TTC TCT GTG	CTA TGG CTT TGA TGG ACT GGA	138
Itgax	XM_574569	ATC TGT GTA AGT GAT GCT ACC C	CTG CTT TCT ACT GGA CTC ATC G	127
CD163	NM_001107887	CTG AAA TCC TCG GGT TGG CA	TGT AGC TGT GGT CAT CCG TG	93

* denotes the endogenous reference genes.

Bac, β-actin; *Hrpt1*, hypoxanthine phosphoribosyltransferase 1; *Sdha*, succinate dehydrogenase complex flavoprotein subunit A; *iNOS*, inducible nitric oxide synthase; *p47*, NADPH oxidase $p47^{phox}$ subunit; *Chi3l1*, chitinase-3-like protein 1; *Itgax*, integrin alpha X; PBMCs, peripheral blood mononuclear cells.

Supplementary S5: Gelatin zymography of the thoracic aorta

(a)

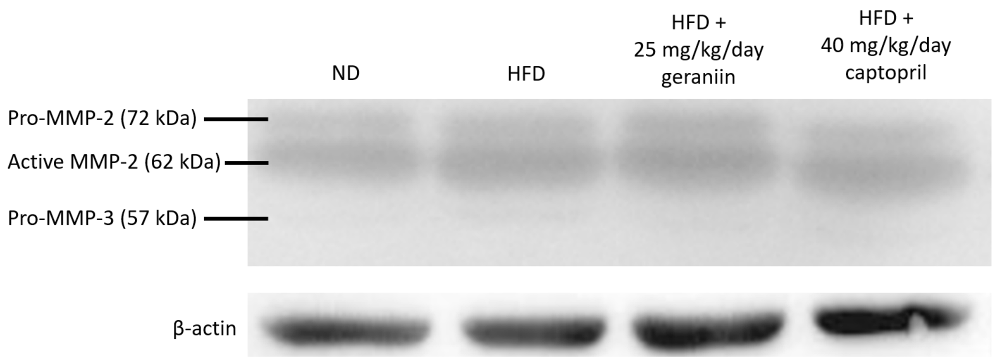

(b)

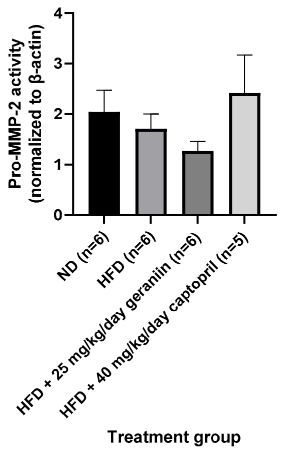

(c)

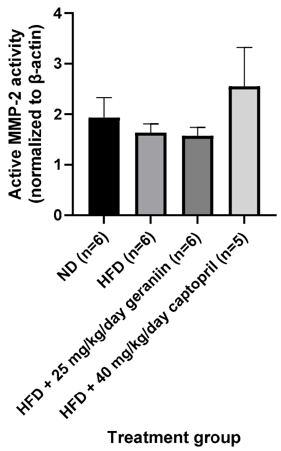

(d)

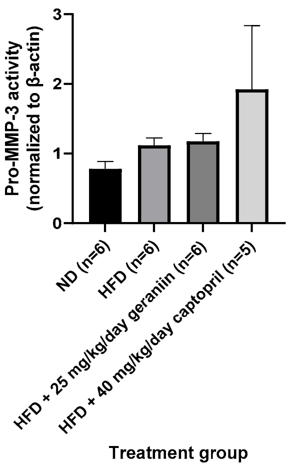

Figure S5. The activities of MMPs in the thoracic aorta of the rats assigned to different treatment groups. (**a**) Gelatin zymography of the thoracic aortae tissue homogenate. The activity of (**b**) pro-MMP-2, (**c**) active MMP-2, and (**d**) pro-MMP-3 in the thoracic aorta. Data are expressed as mean ± SEM with n = 5-6. MMPs, matrix-metalloproteinases; ND, normal diet; HFD, high-fat diet.
